# Localization of Eosinophilic Esophagitis from H&E stained images using multispectral imaging

**DOI:** 10.1186/1746-1596-6-S1-S2

**Published:** 2011-03-30

**Authors:** Pinky A Bautista, Yukako Yagi

**Affiliations:** 1 Department of Pathology, Massachusetts General Hospital, Harvard Medical School Boston, MA,USA

## Abstract

This study is an initial investigation on the capability of multispectral imaging to capture subtle spectral information that would enable the automatic delineation between the eosinophilic esophagitis and other eosin stained tissue components, especially the RBCs. In the method, a principal component analysis (PCA) was performed on the spectral transmittance samples of the different tissue components, excluding however the transmittance samples of the eosinophilic esophagitis. From the average spectral error configuration of the eosinophilic esophagitis transmittance samples, i.e. the difference between the actual transmittance and the estimated transmittance using *m* PC vectors, we indentified two spectral bands by which we can localize the eosinophils. Initial results show the possibility of automatically localizing the eosinophilic esophagitis by utilizing spectral information.

## Background

Eosinophils are type of white blood cells that are important part of the immune system. They are present in small amount in the intestine and blood but not normally in the esophagus. Infiltration of eosinophils into the esophagus could result to conditions such as eosinophilic esophagitis (EE) and gastroesophageal reflux. Since eosinophilic esophagitis and gastroesophageal reflux exhibit similar clinical and histology features studies have been conducted to determine the distinguishing features between the two conditions [1-3] where the number of eosinophil infiltration was found to be one of the indicative features of eosinophilic esophagitis. In [3] pathologists followed three methods to evaluate the infiltration of eosinophils: (1) subjective evaluation of the presence of eosinophils in the entire hematoxylin and eosin (H&E) stained histology section where semi-quantitative scoring was applied; (2) the eosinophils were counted in 5 high power fields (HPF, x400) and the average was calculated; and (3) eosinophil density was evaluated by counting the eosinophils in the mucosa of the entire histological section and measuring the area. The consistency of the results in the subjective and manual approach of assessing the degree the eosinophils infiltration in the methods just mentioned can be improved if appropriate digital processing is applied to the images.

Using the spectral colour of an image pixel for automatic object classification or segmentation can be considered simple if only there is an obvious spectral colour difference between the objects of interest and the background objects. From an H&E stained tissue slide the eosinophils appear red to pink similar to other connective tissue components such as the red blood cells (RBC). Thus there is a challenge in using the RGB colour vector as feature variable for the classification or segmentation of eosinophils.

Multispectral imaging is popularly applied to remote sensing applications, but it has gained significant attentions from researchers in various fields. The technology has been studied for accurate colour reproduction [4], colour enhancement [5,6], digital staining [7], and others. A multispectral imaging system employs more than 3 (N>3) narrowband filters which result to greater spectral sensitivity compared to the conventional RGB imaging system which utilizes 3 broadband filters. The capability of multispectral imaging to delineate tissue structures that are closely similar in their spectral colour have been shown in [8]. In this paper we proposed a method to effectively visualize, detect and segment the eosinophils using information from the multispectral images of H&E stained tissue images, particularly using the spectral error between the original spectral transmittance of a pixel and its estimated transmittance which is calculated by using *m* PC vectors.

## Materials and methods

### Imaging system

**Figure 1:**
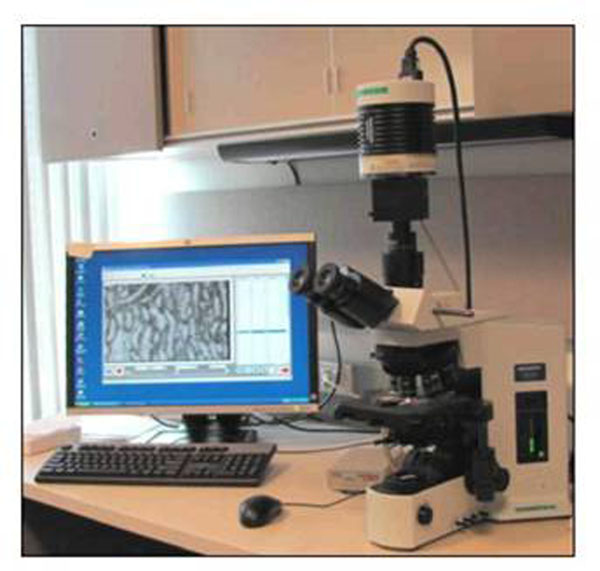
The microscopic multispectral imaging system that was used to capture the multispectral images of H&E stained esophagus tissue in the experiment. Attached to the conventional microscope is a multispectral filter with sensitivity from 400nm to 720nm.

The microscopic multispectral imaging system that we used in our experiment to capture the H&E stained multispectral images is shown in fig. 1. Attached to the conventional microscope is a multispectral filter with spectral sensitivity in the visible spectrum, i.e. 400nm-700nm. Using our in-house software we can capture multispectral images at 5nm bandwidth across the visible spectrum and saved the 1034x1050 pixel images in TIFF format with 16-bit colour depth.

### Multispectral images

We captured 10 sets of H&E stained images with the microscope objective lens set at 20x. In [6,8] 16 multispectral bands were used to enhance regions of collagen fiber to differentiate them from muscle fiber wherein the two tissue structures share similar colorimetric attributes in an H&E stained image. In our experiment the original 55-band multispectral (MS) images were reduced to 14-band MS images by taking the spectral average of the MS pixel at four contiguous bands. Table 1 illustrates the spectral sensitivity of the resulting bands.

**Table 1:** The multispectral (MS) band numbers and their wavelength counterparts. The value of the spectral transmittance at MS band #1 corresponds to the average transmittance value between *λ*_1_ and *λ*_2_ nm.

Multispectral band #	Wavelength [nm]
**1**	**450 - 465**
**2**	**470 - 485**
**3**	**490 - 505**
**4**	**510 - 525**
**5**	**530 - 545**
**6**	**550 - 565**
**7**	**570 - 585**
**8**	**590 - 605**
**9**	**610 - 625**
**10**	**630 - 645**
**11**	**650 - 665**
**12**	**670 - 685**
**13**	**690 - 705**
**14**	**710 - 720**

### Spectral transmittance

The *n*-band spectral transmittance of a pixel is calculated by taking the ratio between the signal value, **i**_s,_ of the specimen and that of glass, **i**_g_:(1)

where the entries of the Nx1 column vector **f** correspond to the spectral values at different wavelengths. We manually extracted the spectral samples for nucleus, cytoplasm, red blood cells (RBC), fiber, white area (the area in the image which is void of tissue) from the 5 images of the 10 sets of images that we captured. .

### Principal component analysis (PCA)

The goal of PCA is to reduce the number feature variables while preserving the variance of the feature data. If we let **F** be an *nxq* data matrix representing *q* samples of an *n*-dimensional feature vector:(2)

then *nxn* covariance of the of the matrix **F** can be derived as follows[9]:(3)

where  is the mean vector of matrix **F** given by:(4)

The diagonal elements of the covariance matrix, c_ii_ , denotes the variance of the data around the mean while the off diagonal elements, c_ij_ , express the degree of correlation between the *ith* and *jth* feature variables. From the covariance matrix **C** we can derive the eigenvectors and eigenvalues and by arranging the eigenvectors in descending order of their eigenvalues we can form an orthogonal basis having the first eigenvector containing the largest variance of the original data. The % variance of the original data using the first *m* eigen vectors can be explained by the ratio between the sum of *m and n* eigenvalues:(5)

where *λ_i_* is the *ith* eigenvalue. If most of the variance in the original data is contained in the first *m* eigenvectors, say 99.99%, a reconstruction of the original data can be done by forming a linear combination of these vectors:(6)

where *α*_i_ is the PC coefficient and **v**_i_ is the *ith* eigenvector.

### Spectral error

The reconstruction error that results from the application of **eqn. 6** largely depends on the accurate estimation of the data covariance matrix **C** which in turn is governed by the sufficiency of the data samples in **F.** If the feature variance of a sample is captured in the data matrix **F** fewer eigenvectors are needed to obtain smaller error in the reconstruction of such sample feature.

PCA is also a technique used to address the estimation of *n* dimensional spectral data by using *m*<*n* eigenvectors. If we consider **F** as the data matrix representing the spectral samples of some objects such that **f**_k_ represents the spectrum of a particular object and  its estimate derived from **eqn. 6** then we can write the spectral error as follows:(7)

where **e**_k_ is an *n* dimensional column vector. The magnitude of the error in **eqn.7** is a function of the estimation of .Consider that there are *c* classes of objects that are identified from an image but only *c-1* of these classes are represented with spectral samples in the data matrix **F,** then for a given *m* eigenvectors the spectral errors are smaller for objects that belong to the first *c-1* classes compared to objects that belong to the *cth* class.

### Detection and segmentation using the spectral error

The configuration of **e**_k_ ,i.e. the wavelengths at which the error peaks, is a function of the spectral attributes of the object. If we could identify two wavelengths *r* and *s* at which the spectral error of the *cth* class has the highest positive and negative peaks while the *c-1* classes of objects experience an almost zero spectral error at these wavelengths it is possible to segment the objects in *cth* class by taking the difference between the spectral errors at these wavelengths:

(8) d = e_r_ – e_s_

where e_r_ is the highest positive peak and e_s_ the negative peak with the largest magnitude. To segment objects belonging to the *cth* class a threshold can be applied to the result of **eqn. 8**(9)

Although in an H&E stained slide the tissue structures are generally categorized as either acidophilic or basophilic each tissue structure has its own distinct spectral attributes due to its unique reactions to the chemical dyes. Hence the spectral error of a tissue component not represented in the data matrix **F** would likely exhibit peaks at certain wavelengths for a given *m* eigenvectors; these wavelengths might be correlated to the absorption peaks of the dyes themselves. With these specific wavelengths identified it is possible to detect and segment such particular tissue component by applying appropriate thresholds. Furthermore translating the spectral error values at these wavelengths would also result to better visualization of the tissue component.

**Figure 2:**
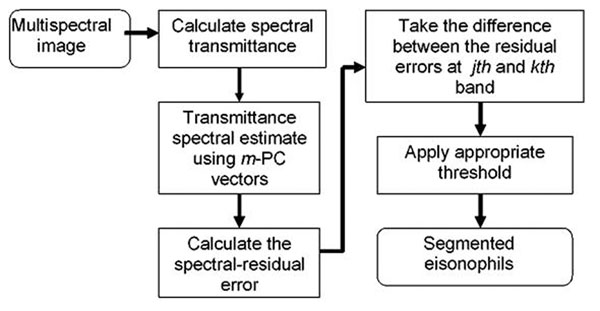
Block diagram showing the processes involve in the segmentation of eosinophils

The general processes involved in the localization of eosinophils are illustrated in the block diagram in fig.2. First the spectral transmittance of a pixel is calculated then its estimate is calculated using *m* PC vectors that were previously identified in an off line experiment. The difference of the spectral errors between two bands is then calculated. Application of an appropriate threshold, **eqn.9**, segments the eosinophils.

## Results

The objective of this paper is to detect and segment the eosinophils from H&E stained esophagus tissue images. For the purpose of the experiment we collected 10 multispectral images from two different H&E stained slides.

### Spectral residual-error

**Figure 3:**
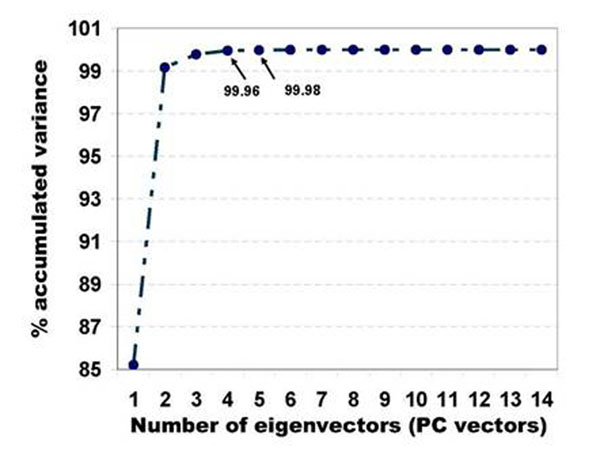
The % accumulated variance for different number of eigenvectors (PC vectors). This plot has been generated using the spectral samples of nuclei, cytoplasm, red blood cells, fiber and white areas (areas which do not contain tissue). We can observe that with more number of PC vectors the variance of the data samples is captured better.

The training set **F** consisted of over 3000 transmittance samples belonging to five tissue components: nucleus, cytoplasm, red blood cells (rbc), fiber and white area (area which does not contain any tissue). From this data set we calculated the covariance matrix **C** and derived the eigen vectors or principal component (PC) vectors, and the eigenvalues. The plot in fig.3 shows the % accumulated variance calculated from **eqn. 5**. It can be interpreted from the plot that with 5 PC vectors the spectral errors of the five tissue components would reduce to zero.

**Figure 4:**
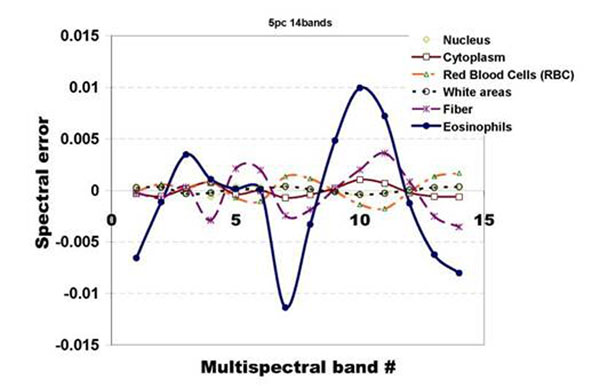
The graph shows the almost zero average spectral-errors of the 5 tissue components whose spectral samples were used to calculate the covariance matrix. The average spectral error of the eosinophils exhibit peaks at specific wavelengths, i.e. band7 -570nm-585nm; band 10-630nm-645nm.

The graph in fig.4 illustrates the configurations of the 14-band average spectral errors of the 5 tissue components whose spectral samples were used to derive the covariance matrix that of eosinophils. We see from the graph that the exclusion of the eosinophil spectral samples from the training data results to larger spectral error magnitude and distinct peaks at certain wavelengths. For instance at band 7 (570nm-585nm) and band 10(630nm-645nm) largest negative and positive peaks are observed.

### Detection and Segmentation of the eosinophils

**Figure 5:**
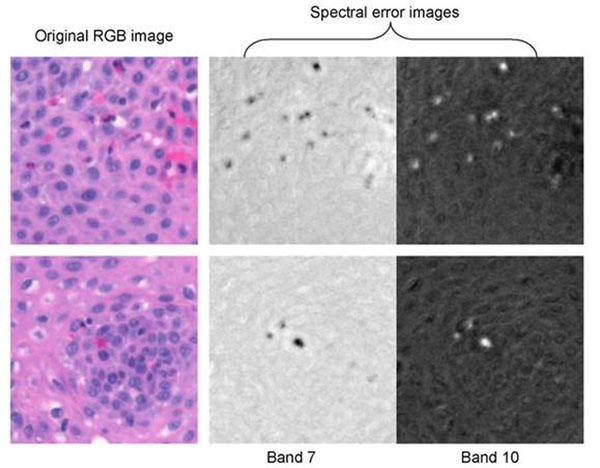
Original H&E stained images in RGB color format along with the grey-level images of the spectral errors at band 7 and band 10. The spectral error images show better visualization of the eosinophils wherein the darkest spots and whitest spots in band 7 and band 10 images correspond to eosinophils, respectively.

**Figure 6:**
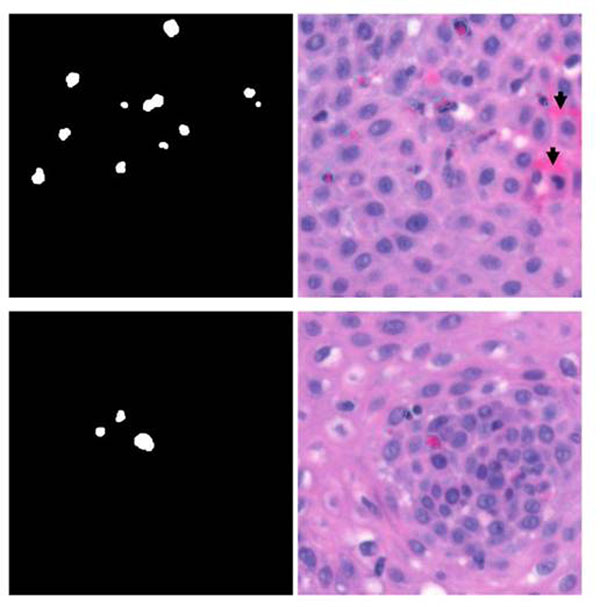
Segmentation results using multispectral information. The segmentation was done by taking the difference between the spectral errors at band 7 and band 10 and then applying the threshold specified by eqn.10; the images on the 2^nd^ column show the segmentation results overlaid on the original RGB images. It is worth noting that tissue structures (indicated by arrows) which acquired similar colorimetric attributes to eosinophils were not identified as eosinophils.

The wavelengths (or bands) at which the spectral errors of the eosinophils are the largest in magnitude were identified from the spectral-error plot itself, fig.5. After we have identified these wavelengths we converted the error values at these wavelengths to grey-level image to obtain better visualization of the eosinophils. Samples of the H&E stained images, which were extracted from an esophagus tissue slide containing eosinophils, in their RGB format along with spectral error images at band 7 (570nm-585nm) and band 10 (630nm-645nm) are shown in fig.6. The eosinophils are stained pink to red, which is similar with other connective tissues such as the red blood cell. However from the error images such similarity is not visible – the eosinophils are distinctly marked in the images. In band 7 the eosinophils appear as black spots because they acquired the lowest spectral error at this band, refer to fig.5. Since the spectral error of the eosinophils at band 10 is the highest they are the white spots in the error image at this band.

To segment the eosinophils the spectral errors at band 7 and band 10 were utilized. By implementing **eqn. 8 and 9** where e_r_ correspond to the spectral error at band 10 and e_s_ to the error at band 7, the eosinophils were successfully segmented. Figure 6 shows the result of the segmentation after applying morphological filter, i.e. dilation and erosion (edge detection using the canny algorithm as implemented in Matlab was applied to the results before they are overlaid on the original RGB color images). Here we see that the method was able to delineate between eosinophils and other similarly stained tissue components, i.e.RBC. There are however few eosinophils that were not detected by the process and this can be because (i) the spectral training samples was insufficient; (ii) the bandwidth of each spectral band is not narrow enough to capture minute changes in the spectral colours; and/or (iii) the number of spectral bands is not enough to capture the subtle spectral difference between eosinophils and other eosin stained tissue structures.

### Segmentation using RGB color information

**Figure 7:**
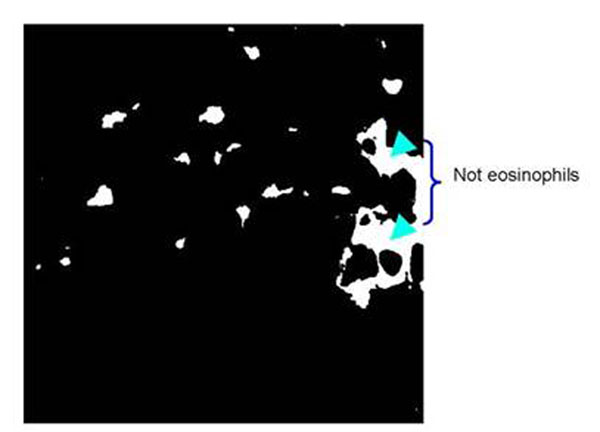
Segmentation result by *k*-means (*k*=*6*) clustering using the RGB color information showing the cluster group containing the eosinophils.If we refer this result to the original RGB color image (fig.7, topmost, 2^nd^ column) we can we see that tissue structures which are not eosinophils at all were also identified as eosinophils in the result, particularly the areas indicated by arrows.

To determine the effectiveness of using RGB color information to segment the eosinophils we implemented a *k*-means clustering to the image containing both eosinophils and RBC. We set *k* =6 to represent all the tissue components that we have identified from the training images and this includes the eosinophils. The eosinophil areas that result from the clustering are shown in fig. 7. Here we see that clustering the image pixels using the RGB color information failed to detect the difference between the eosinophils and the red blood cells; the mis-classified areas are indicated by the arrows.

## Discussion

In a stained tissue slides, tissue structures which differ in their functionality can be stained with the same dye. The eosinophils and red blood cells (RBC), for instance, are both stained with eosin dye which made them share similar color attributes, i.e. pink to red. We have introduced a simple way to detect and segment the eosinophils using multispectral information particularly from the spectral errors of the multispectral pixels. The utilization of spectral errors was first introduced in [5] to enhance abnormal skin areas. With modifications of the method the used of spectral error was applied to H&E stained images to improve the colorimetric difference between collagen and muscle fiber [6,7]. Spectral error subtraction as a method to segment tissue components, which is currently being utilized to segment/detect eosinophils, was not addressed in both papers, however.

**Figure 8:**
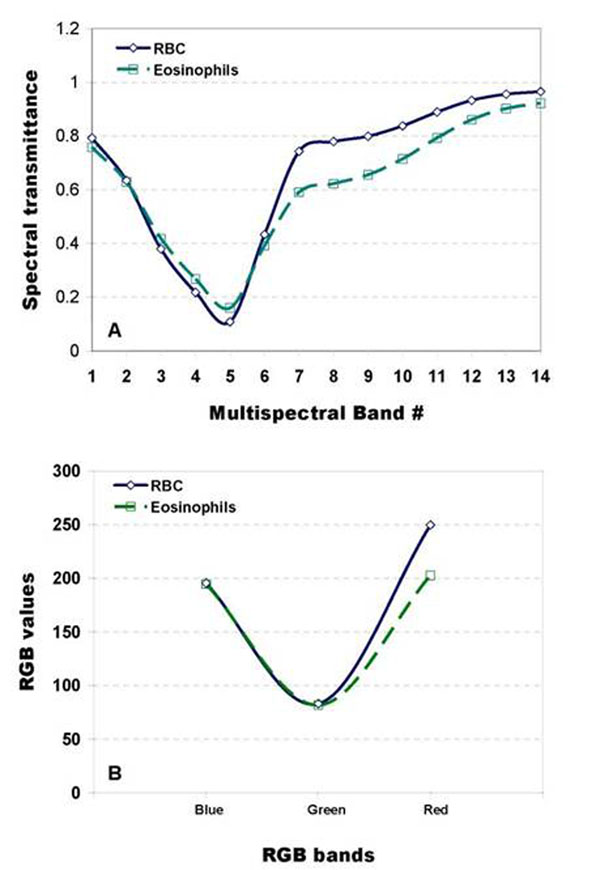
Panel A and Panel B show graphs of the characteristic 14-band spectral transmittance and 3-band (RGB) spectral colour of the eosinophils and RBC (red blood cells), respectively. In panel A we can see differences between the RBC and eosinophil in their spectral configurations but their RGB colour values do not significantly differ as illustrated in Panel B, except in their red colour intensity which can vary depending on the staining.

The effectiveness of utilizing multispectral information as opposed to RGB color information was shown in the segmentation results where the results using only the RGB color information was found inferior to the results from using multispectral information. An average plot of the 14-band spectral transmittance and RGB color values of 20 RBC and eosinophil samples are shown in fig.8. While we can see variations between the spectra of RBC and eosinophil in the whole visible spectrum, this is not directly observed from the RGB color plots where the difference is concentrated in the long wavelength range, i.e. red channel. That is the two structures likely differ in their red color intensity in which case minute shift in the color cannot be detected.

**Figure 9:**
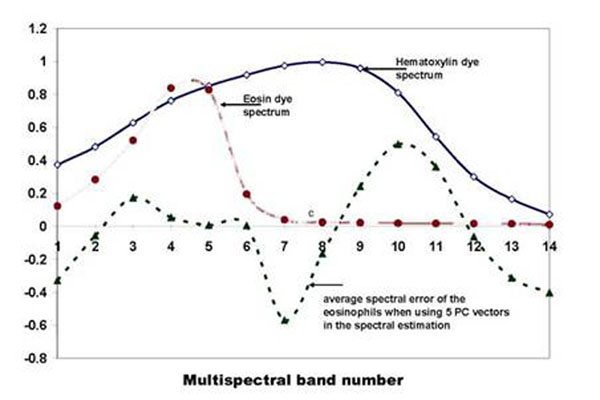
The eosin and hematoxylin dye absorption spectra and the average spectral error of the eosinophils; the spectral error of the eosinophils is scaled to have a clearer illustration. We can observe that the error peaks of the eosinophils correspond to the absorption peak of the hematoxylin dye. This could imply that the difference between eosinophils and other tissue structures which are stained similarly lies on their absorption of the hematoxylin dye.

The peaks exhibited by the spectral error of eosinophil in fig.4 can be correlated to the dye absorption peaks. Plots of the hematoxylin and eosin 14-band dye absorptions are shown in fig. 9; the spectral error of the eosinophil is scaled and superimposed on the plot. We can observe that the peaks at band 7 and band 10 correlate to the hematoxylin absorption peak. The correspondence of the spectral error peak to the absorption peak of hematoxylin dye could imply that the difference between RBC and eosinophils can be accounted to their absorption of hematoxylin dye. More experiments however, are still needed to further justify this claim.

## Conclusions

In this paper we have addressed the detection and segmentation of eosinophils from an H&E stained slide of an esophagus tissue using multispectral information, particularly using the spectral error difference between two specific bands that were identified from the plot of the spectral error itself. The results of the experiment show that with multispectral information it could be possible to classify tissue structures with very similar staining attributes which is difficult with the conventional RGB color information.

Quantification of eosinophils in H&E stained esophagus tissue images is helpful in identifying eosinophilic esophagitis from gastroesophageal reflux. The segmentation of eosinophils presented in this paper can be utilized as a first step in the quantification process. Moreover, the proposed method could also be useful to segment other tissue structures which are stained similarly by simply excluding the spectral samples of such structures in the derivation of the eigenvectors (PC vectors) that are used to estimate the spectral transmittance of a multispectral pixel.

## Authors' contributions

The authors contributed equally.

## Competing interests

MGH received financial grant from Olympus Co., Japan, but not for this project.
